# Isolation and characterization of pathogenic *Escherichia coli* bacteriophages from chicken and beef offal

**DOI:** 10.1186/s13104-019-4859-y

**Published:** 2020-01-06

**Authors:** Celosia Lukman, Christopher Yonathan, Stella Magdalena, Diana Elizabeth Waturangi

**Affiliations:** 1 Department of Food Technology, Faculty of Biotechnology, Atma Jaya Catholic University of Indonesia, Jakarta, 12930 Indonesia; 2 Department of Biology, Faculty of Biotechnology, Atma Jaya Catholic University of Indonesia, Jakarta, 12930 Indonesia

**Keywords:** Bacteriophages, Beef offal, Biocontrol, Chicken, *Escherichia coli*, Foodborne pathogen

## Abstract

**Objective:**

This study was conducted to isolate and characterize lytic bacteriophages for pathogenic *Escherichia coli* from chicken and beef offal, and analyze their capability as biocontrol for several foodborne pathogens. Methods done in this research are bacteriophage isolation, purification, titer determination, application, determination of host range and minimum multiplicity of infection (miMOI), and bacteriophage morphology.

**Results:**

Six bacteriophages successfully isolated from chicken and beef offal using EPEC and EHEC as host strain. Bacteriophage titers observed between 10^9^ and 10^10^ PFU mL^−1^. CS EPEC and BL EHEC bacteriophage showed high efficiency in reduction of EPEC or EHEC contamination in meat about 99.20% and 99.04%. The lowest miMOI was 0.01 showed by CS EPEC bacteriophage. CI EPEC and BL EPEC bacteriophage suspected as *Myoviridae* family based on its micrograph from Transmission Electron Microscopy (TEM). Refers to their activity, bacteriophages isolated in this study have a great potential to be used as biocontrol against several foodborne pathogens.

## Introduction

*Escherichia coli* had been reported as fundamental source of many foodborne illness cases. Food preservation to prevent foodborne illness usually done by physical or chemical method. However, these methods could reduce the food’s quality and organoleptic properties. Addition of chemical agents like antibiotics is still a concern because it can develop antibiotic resistant bacteria [1].

Bacteriophages is a natural alternative method for preserving foods because they are viruses that can target bacteria specifically without disrupting human, plant, or animal cell [2]. In recent years, usage of bacteriophages as biocontrol had been increasing because of its specificity, self-replicating ability, and abundant in food [3]. Bacteriophage must be lytic and non-transducing as minimum requirements to ensure safety [4].

Chicken and beef are the most consumed meat worldwide and has been identified as main reservoir of foodborne pathogenic bacteria [5]. Chicken and beef offal have high protein content, therefore they can be easily contaminated by bacteria [6]. The objectives of this research were to isolate and characterize lytic bacteriophages for pathogenic *E. coli* from chicken and beef offal and analyze their capability as biocontrol for several foodborne pathogens.

## Main text

### Methods

#### Inoculum preparation

*Escherichia coli* ATCC 25922, Enteropathogenic *E. coli* (EPEC), and Enterohaemorrhagic *E. coli* (EHEC) from Atma Jaya culture collections were used in this study. Bacterial cultures were inoculated onto LB Agar and incubated at 37  °C overnight.

#### Sample collection

Beef offal (lung, intestine, and liver) and chicken samples (skin and intestine) were randomly picked as sample of the offal and purchased from traditional markets in Tangerang, Indonesia.

#### Bacteriophage isolation

Each bacterial strain was grown in LB Broth to mid-log phase by incubation at 37 °C and 120 rpm. Samples were suspended 1:10 (w/v) in SM buffer and stomached, followed by bacterial addition to ratio of 9:1 (v/v) [7]. Samples were supplemented with 10 mM CaCl_2_ and 0.5 mM MgSO_4_ to enhance bacteriophage growth [8]. Enriched samples were incubated at 37 °C and 120 rpm overnight and centrifuged at 8624×*g* for 10 min. The supernatant was filtered with a 0.45 μm pore-size disposable syringe filter (Axiva, Faridabad, India) to remove remaining bacterial cells. The filtrate was then examined for the presence of bacteriophages using agar overlay assay [9]. Filtrates that formed plaques were stored at 4 °C on SM buffer and used as bacteriophage lysate solution for further analysis [7, 10].

#### Bacteriophage purification and enrichment

Sterile tip gently stabbed in the center of the plaque. The tip was then placed into 5 mL of LB Broth and pipetted up and down to release bacteriophage particles. About 250 μL bacterial host was then added into LB Broth. Bacteriophage was enriched for 6 h at 37 °C and 120 rpm, then centrifuged at 8624×*g* for 10 min and filtered with a 0.45 μm syringe filter. A tenfold serial dilution was made and plated according to agar overlay assay. This process was repeated until all plaque morphologies were consistent [10, 11].

#### Bacteriophage titer determination

A series of tenfold dilutions in SM buffer were made of bacteriophage lysate solution and plated according to agar overlay assay. The number of visible plaques were counted between 30 and 300 plaques which expressed as PFU mL ^− 1^ [7].

#### Bacteriophage application

Meats were cut into thin 1 × 1 cm squares and sterilized for 15 min at 121 °C. EPEC or EHEC was inoculated 10^4^ CFU cm ^− 2^ onto meat’s surface, followed by 10^8^ PFU mL ^− 1^ of diluted phage suspension. Meats were incubated at 25 °C overnight. For negative control, same volume of SM buffer was used instead of phage suspension [12]. Meats were suspended with 9 mL of SM buffer. A tenfold serial dilution was made then spreaded onto LB Agar and incubated at 37 °C  overnight. Colonies were counted between 30 and 300 colonies which expressed as CFU mL^−1^ [12].

#### Host range determination

EPEC, EHEC, ETEC, and *E. coli* ATCC 25922 were tested for their susceptibility to isolated bacteriophages. Bacteriophage stock solution was pipetted 100 μL into 3 mL top agar with addition of 100 μL of tested bacteria, then poured into bottom agar and incubated aat 37 °C overnight [13].

#### Efficiency of plating (EOP)

Positive result bacteriophage on host range determination was diluted and tested using agar overlay assay. When the dilution did not result any plaques, a lower dilution was tried afterwards to verify the lower EOP. EOP was calculated by dividing the average PFU on target bacteria with average PFU on host bacteria [14].

#### Minimum inhibitory multiplicity of infection (miMOI)

EPEC and EHEC were grown to mid-log phase and suspended to match 0.132 McFarland standard, then diluted until 10^5^ CFU mL^−1^ and distributed 100 μL into the 96-well microtiter plate. Bacteriophages were diluted to contain different MOI from 0.00001 to 100 and 100 μL was added to wells containing bacterial cells. Plate was incubated at 37 °C for 10 h and bacteriophage’s concentration determined every 1 h [15].

#### Bacteriophage morphology

Bacteriophage’s morphology was determined using Transmission Electron Microscopy (TEM) at the Eijkman Institute, Jakarta, Indonesia. About 10 μL of bacteriophage was dropped on grid (400mesh) and left for 30 s. Bacteriophage samples were negatively stained using 5 μL of 2% (w/v) uranyl acetate on carbon-coated grids. The grids were observed using JEM-1010 TEM (JEOL, Tokyo, Japan) at magnification of  × 30,000 [16].

#### Statistical analysis

A one-way ANOVA (SPSS Inc. IBM corporation) and Tukey’s-B test were done to indicate any significant difference of bacterial concentration between control and bacteriophage treatment. Level of significance was defined at P ≤ 0.05.

### Results

#### Bacteriophage isolation

A total of six bacteriophages were isolated in this study. Bacteriophages that lyse EPEC were found in chicken intestine (CI), chicken skin (CS), beef lung (BL), and beef intestine (BI), while bacteriophage that lyse EHEC only found in BL and BI. All positive results were denoted as lytic bacteriophages due to the clear zone plaques on agar.

#### Bacteriophage titer determination

Bacteriophage titers observed between 10^9^ and 10^10^ PFU mL ^− 1^ (Table 1). The highest titer of bacteriophage was isolated from BI EPEC bacteriophage with a titer of 2.62 ± 0.67 × 10^10^ PFU mL ^− 1^.

**Table 1:** Titer and effectivity of bacteriophages

Bacteriophage	Titer (PFU mL ^− 1^)	Bacteriophage application				Efficiency of plating			
		Control (CFU mL ^− 1^)	Bacteriophage treatment (CFU mL ^− 1^)	Bacteria reduction (log)	Bacteria reduction (%)	EPEC	EHEC	ETEC	EC
CI EPEC	2.30 ± 0.50 × 10^9^	2.11 ± 0.4 × 10^8 a^	9.05 ± 1.20 × 10^7 ab^	0.37	57.15	*1.0*	–	0.02 ± 0.004	–
CS EPEC	1.60 ± 0.40 × 10^10^	2.11 ± 0.4 × 10^8 a^	1.68 ± 0.32 × 10^6 b^	2.10	99.20	*1.0*	–	0.1 ± 0.006	–
BL EPEC	1.39 ± 0.28 × 10^10^	2.11 ± 0.4 × 10^8 a^	1.35 ± 0.34 × 10^7 b^	1.19	93.61	*1.0*	0.0004 ± 0.0003	0.52 ± 0.14	0.47 ± 0.01
BI EPEC	2.62 ± 0.67 × 10^10^	2.11 ± 0.4 × 10^8 a^	1.06 ± 0.08 × 10^7b^	1.30	95.00	*1.0*	0.03 ± 0.01	0.28 ± 0.16	0.11 ± 0.05
BL EHEC	1.43 ± 0.39 × 10^10^	1.25 ± 0.46 ×10^9 a^	1.20 ± 0.20 x 10^7 a^	2.02	99.04	–	*1.0*	3.87 ± 0.95	–
BI EHEC	1.63 ± 0.46 × 10^10^	1.25 ± 0.46 × 10^9 a^	1.12 ± 0.11 × 10^8 a^	1.05	91.02	–	*1.0*	1.08 ± 0.63	–

^a,b^Means in the same row having different superscript are differ significantly (P ≤ 0.05). Data were shown in mean ± standard error

#### Bacteriophage application

Cooked meat was artificially contaminated with host bacteria to calculate bacteria reduction if samples were added with bacteriophages. Samples without bacteriophages added were used as negative control. CS EPEC, BL EPEC, and BI EPEC bacteriophage significantly reduced bacterial concentration in meat samples. However, no significant reduction was observed when samples added with CI EPEC, BL EHEC, and BI EHEC bacteriophage. The results are shown in Table 1.

#### Host range determination and efficiency of plating

The host range of each bacteriophage was determined against EPEC, EHEC, ETEC and *E. coli* ATCC 25922. The efficiency of plating of each bacteriophage are shown in Table 1. The plating on original strain of isolation (EOP = 1.0) was marked in italic.

#### Minimum inhibitory multiplicity of infection (miMOI)

**Fig. 1:**
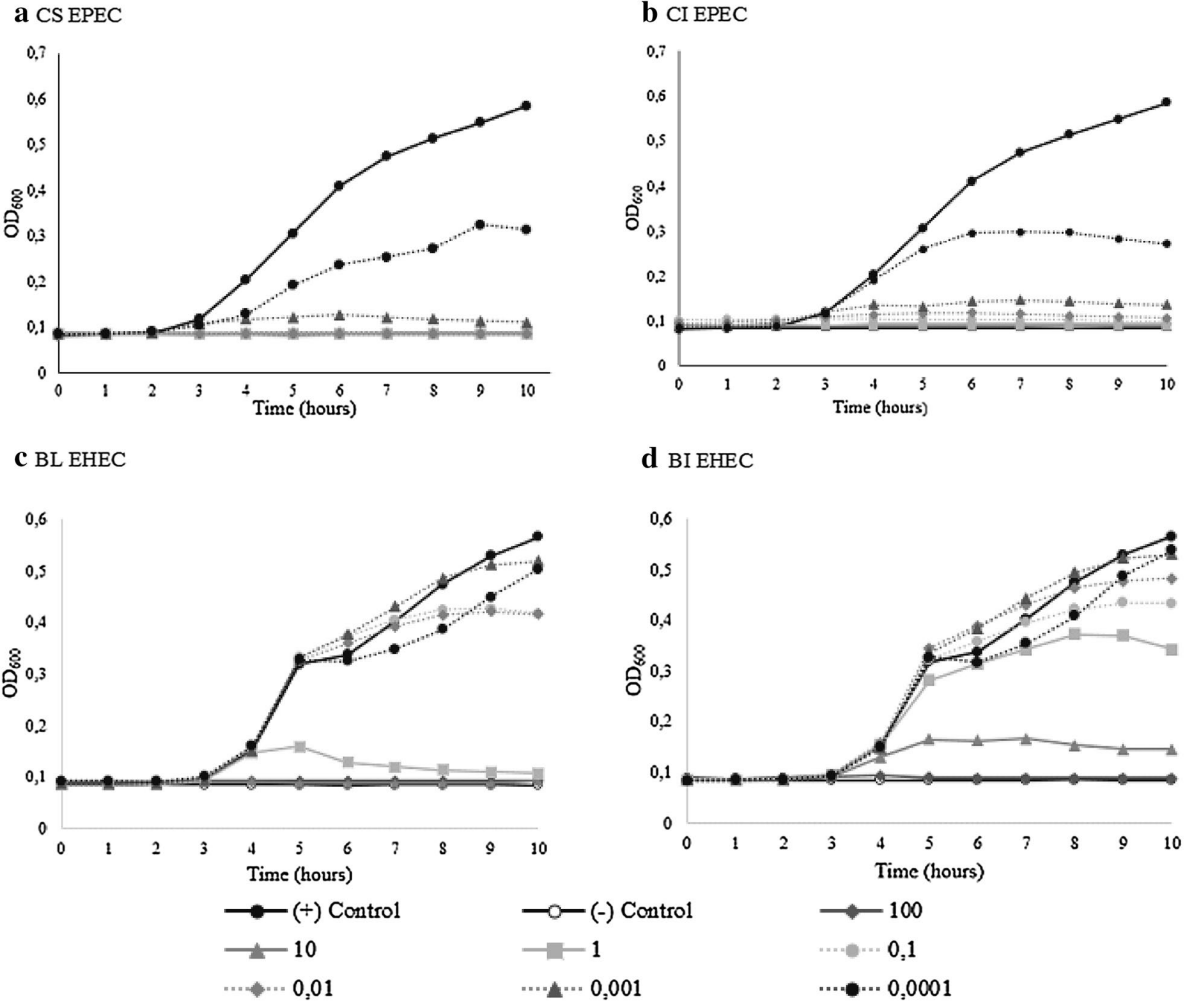
The effect of different MOI of bacteriophages to host bacteria’s growth

The miMOI of CS EPEC bacteriophage observed was 0.01, while CI EPEC bacteriophage and BL EHEC bacteriophage were 1, and BI EHEC bacteriophage was 100. Bacterial growth was increasing as the MOI decreased (Fig. 1).

#### Bacteriophage morphology

**Fig. 2:**
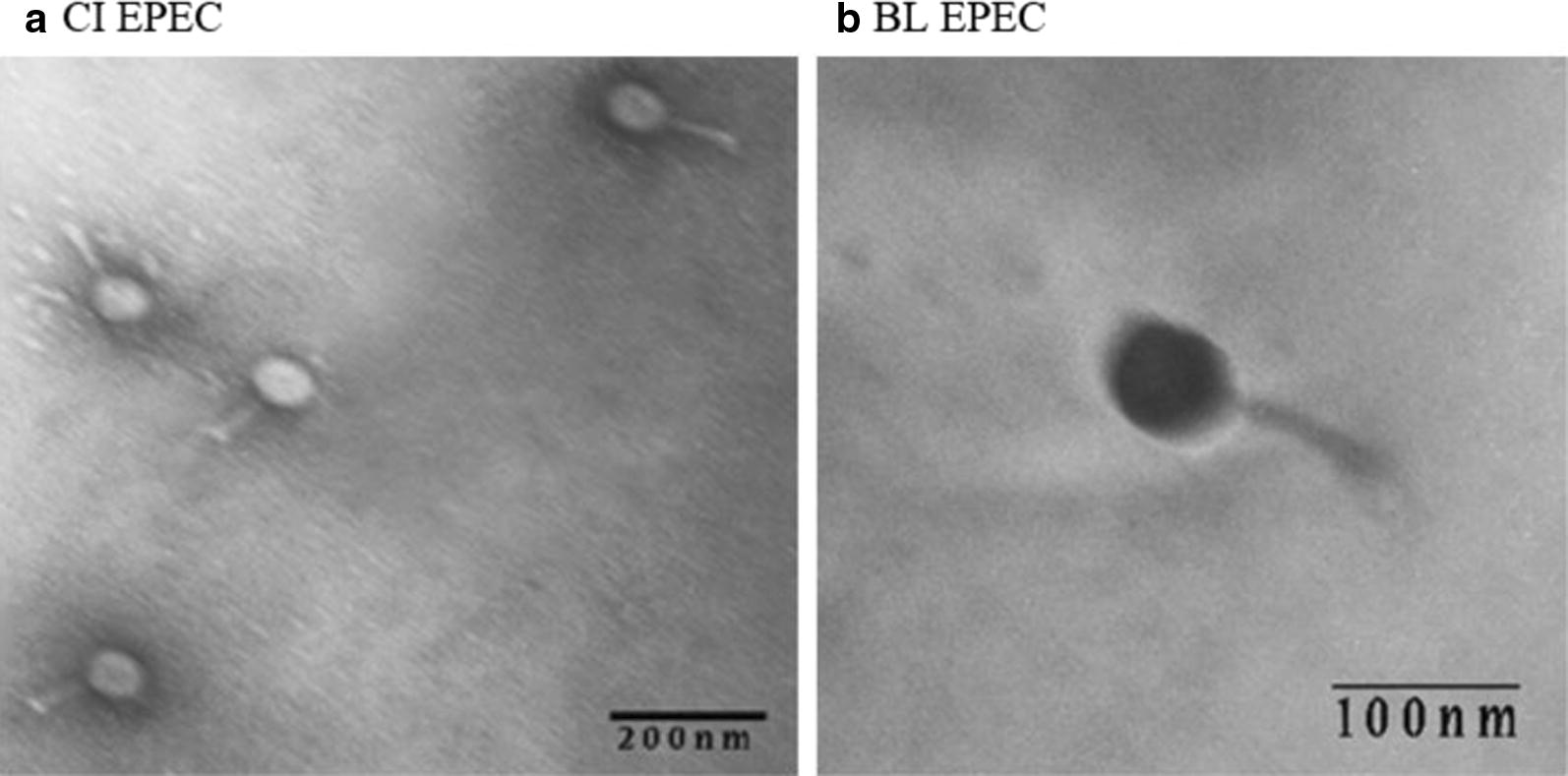
Electron micrographs of negatively stained bacteriophage

Bacteriophage morphology was determined for CI EPEC and BL EPEC bacteriophages. TEM showed that both bacteriophages had an icosahedral head and a contractile tail (Fig. 2). The tail length was about 83 nm for CI EPEC bacteriophage and 90 nm for BL EPEC bacteriophage, while diameter of the head was about 67 nm for CI EPEC bacteriophage and 70 nm for BL EPEC bacteriophage.

### Discussion

In this study, a total of six bacteriophages were successfully isolated from chicken and beef offal samples with EPEC and EHEC as their host. Isolated bacteriophages were indicated as lytic bacteriophage due to the clear plaques on agar [13]. However, no *E. coli* ATCC 25922 bacteriophage was found and no bacteriophage was recovered from beef liver and beef meat samples. This could be due to the adaptation to different environment and interaction of biotic and abiotic components [7].

Bacteriophage titers were observed between 10^9^ and 10^10^ PFU mL ^− 1^. Other study showed a similar result with bacteriophage titers observed between 10^8^ and 10^11^ PFU mL ^− 1^ [7]. Bacteriophage titers variation could be caused by the difference of bacteriophages viability in food samples.

CS EPEC and BL EHEC bacteriophages showed high efficiency in reduction of EPEC or EHEC in meat about 99.20% and 99.04%, respectively. Inactivation of bacteria on food could be affected by food matrix ability to absorb liquid from bacteriophage suspension and distribution of bacteriophage particles [17]. Inadequate nutrition, poor environments (acidity, temperature, and water content), and a switch of host to stationary phase could decrease bacteriophages’ productivity [18, 19].

Broad host range bacteriophages were more desirable to kill multiple species of bacteria when applied to foods [17]. Each bacteriophage had different receptor binding protein (RBP) that can bind to specific receptor on host cell surface. Differentiation between bacteriophage binding in Gram-negative bacteria may be caused by their difference in O-antigen of lipopolysaccharides (LPS) that serve as receptor [20].

Positive results in host range determination were then tested for EOP. EOP was the ratio between bacteriophage titer on tested strain to bacteriophage titer on strain used to isolate the bacteriophage. EOP higher than 0.5 was ranked as high efficiency, 0.2 to 0.5 was medium efficiency, 0.001 to 0.2 was low efficiency, and below 0.001 was not effective [21]. BL EHEC bacteriophage showed highest EOP in ETEC which was 3.87 ± 0.95, while there was no other bacteria except ETEC could be lysed by BL EHEC bacteriophage. This indicate that BL EHEC bacteriophage was highly effective but work specifically on ETEC. CS EPEC and CI EPEC bacteriophage had low efficiency towards ETEC, could not infect EHEC, and *E. coli* ATCC 25922. Low EOP could be caused by host resistance system that blocked virus development, or poor bacteriophage adsorption into host cells [22].

miMOI was estimated as the minimum ratio of bacteriophage and bacteria that completely inhibited the growth of bacterial cells at second hour of stationary phase of positive control [23]. In this study, miMOI was determined for CI EPEC, CS EPEC, BL EHEC and BI EHEC bacteriophages. CS EPEC bacteriophage showed lowest miMOI, indicated that CS EPEC bacteriophage was the most effective because lower concentration of bacteriophage was needed to reduce bacterial growth. Lower MOI could not completely inhibited the growth of the cells, yet bacterial cell is still reduced [17].

According to TEM result, CI EPEC and BL EPEC bacteriophages were indicated as Caudovirales because of its tail. Caudovirales was divided into three families based on the tail morphology. Bacteriophage with long contractile tail was classified as Myoviridae, while Siphoviridae had long non-contractile tail and Podoviridae had short non-contractile tail [24]. Bacteriophage in this study had icosahedral head with long contractile tail of approximately 83 and 90 nm. Based on those properties, we assumed that this bacteriophage could belong to Myoviridae. Further research such as bacteriophage genome sequencing is needed to ensure the bacteriophage classification [25].

### Conclusion

Bacteriophages isolated in this study had a high titer and could effectively reduced bacteria concentration in artificially contaminated cooked meat. Based on morphology determination using TEM, all isolated bacteriophages suspected as Myoviridae according to its long contractile tail. It can be concluded that all isolated bacteriophages in this study have a great potential to be used as biocontrol for food safety.

## Limitation

Isolated bacteriophages not tested to another pathogenic and non-pathogenic bacteria aside from *E. coli*.

## Data Availability

The data of this study is available with the corresponding author up on request.

## Abbreviations

miMOI: minimum inhibitory multiplicity of infection
EOP: efficiency of plating
